# Loop-Mediated Isothermal Amplification Method for the Rapid Detection of *Ralstonia solanacearum* Phylotype I Mulberry Strains in China

**DOI:** 10.3389/fpls.2017.00076

**Published:** 2017-01-31

**Authors:** Wen Huang, Hao Zhang, Jingsheng Xu, Shuai Wang, Xiangjiu Kong, Wei Ding, Jin Xu, Jie Feng

**Affiliations:** ^1^Institute of Plant Protection, Chinese Academy of Agricultural SciencesBeijing, China; ^2^College of Plant Protection, Southwest UniversityChongqing, China

**Keywords:** *Ralstonia solanacearum*, phylotype I mulberry strains, loop-mediated isothermal amplification, detection, bacterial wilt

## Abstract

*Ralstonia solanacearum* phylotype I mulberry strains are causative agent of bacterial wilt of mulberry. Current diagnostic methods are not adopted to the mulberry wilt disease. In this study, we developed a rapid method, loop-mediated isothermal amplification (LAMP), to detect *R. solanacearum* phylotype I mulberry strains. A set of six primers was designed to target the clone MG67 sequence in this LAMP detection which can be completed in 20 min at 64°C. The results of the LAMP reaction could be observed with the naked eye due to magnesium pyrophosphate precipitate produced during the reaction or the color change after adding SYBR Green I. The specificity of the LAMP was confirmed using DNA from 46 representative strains of *R. solanacearum* and 7 other soil-borne bacteria strains. This method was also of high sensitivity and could be used to detect the presence of less than 160 fg genomic DNA or 2.2 × 10^2^ CFU/ml of bacterial cells per 25 μl reaction volume, moreover, the presence of plant tissue fluid did not affect the sensitivity. Since it does not require expensive equipment or specialized techniques, this LAMP-based diagnostic method has the potential to be used under field conditions to make disease forecasting more accurate and efficient.

## Introduction

Bacterial wilt caused by *Ralstonia solanacearum* has an extremely wide geographic distribution in tropical, subtropical and some warm temperate regions of the world, and poses a huge threat to the production of many economically important food and cash crops (Hayward, 1991). *R. solanacearum* is metabolically versatile and can survive not only in soil but also in latently infected plants and water (Xue et al., 2011). The species complex has been subdivided into four phylotypes (Phylotypes I, II, III, and IV) corresponding to the four genetic groups identified via sequence analysis (Fegan and Prior, 2005).

The causative agents of BWM was designated as phylotype I mulberry strains. BWM was initially found in Guangdong province of Southern China (He et al., 1983), and has been discovered as a restricted distribution in Guangxi, Yunnan, Shandong, Jiangxi, and Zhejiang provinces (Pan et al., 2013). Since 2002, this vascular disease has increased severely in Zhejiang province and has been regarded as an important bottleneck in the local sericulture industry. BWM has been dramatically reduced to a low damage level attributed to the implementation of integrated management strategies, i.e., phytosanitation, rotation with corn and use of tolerant cultivars. However, the propagating materials, contaminated by *R. solanacearum* phylotype I mulberry strains, have important impact on re-outbreaks of BWM in some orchards. Therefore, rapid detection and accurate diagnosis of *R. solanacearum* phylotype I mulberry strains in the field is essential to prevent the bacterial dissemination with asymptomatic and symptomless germplasms from poorly controlled areas to well managed ones.

Several diagnostic methods have been widely used to detect *R. solanacearum*. For instance, serological techniques relying on the composition of bacteria (Griep et al., 1998) and molecular techniques that are based on the DNA or RNA of viable bacterial cells (Seal et al., 1993; Weller et al., 2000; Bentsink et al., 2002) have been reported. Furthermore, some PCR-based methods have been developed for subspecies-specific detection (Horita et al., 2004; Prior and Fegan, 2005; Thammakijjawat et al., 2006; Smith and De Boer, 2009; Paret et al., 2010; Ha et al., 2012). However, since the conserved genomic sequence of *R. solanacearum* phylotype I mulberry strains have not been identified, less work has been done on the detection of them. A multiplex PCR method based on the genetic diversity within BWM strains in China was developed in 2013 to specifically detect the mulberry strains (Pan et al., 2013). Although this was a simple and efficient method using the primer set MG67-F/MG67-R in combination with the species-specific primer pair AU759f/AU760r, there were still shortcomings to overcome. It required a high precision thermal cycler for the amplification as well as electrophoresis gel imaging equipment for the determination of the results, which made this method only available in the laboratory and unsuitable in the field. In addition, it could not be used for rapid detection, since it took more than 2 h.

Loop-mediated isothermal amplification (LAMP), first described by Notomi et al. (2000), has the potential to be more suitable for field use than PCR-based methods since it allows the synthesis of large amounts of DNA in a short time and under isothermal conditions ranging from 60 to 65 °C (Tomotada et al., 2003; Tomlinson et al., 2010). As a novel method to amplify nucleic acid, LAMP employing a set of six primers and a *Bst* DNA polymerase with strand displacement activity is characterized by its high specificity, efficiency, and rapidity. (Nagamine et al., 2002).

Loop-mediated isothermal amplification requires a set of four specifically designed primers that recognize six distinct sequences on the target DNA; its specificity is extremely high. The primers essential for the LAMP reaction include a FIP, a BIP, a forward outer primer (F3) and a backward outer primer (B3). FIPs and BIPs, of which both the 3′ region and 5′ region match the target, interact with upstream external primers resulting in the displacement of strands containing self-complementary regions that form stem-loop structures (Tomlinson et al., 2010). Nagamine et al. (2002) developed a method that accelerates the LAMP reaction by using additional primers, termed loop primers. Those primers can also improve the selectivity since they need appropriate starting material for LAMP cycling (Nagamine et al., 2002). The results of LAMP can be observed by the naked eye since the reaction by-product pyrophosphate ions bind to magnesium ions and form a white precipitate of magnesium pyrophosphate (Mori et al., 2001). In addition, the LAMP products can also be directly detected by adding SYBR Green I to the tube and observing the color-change of the solution, which turns green if the LAMP amplification occurs, while it remains orange if there is no amplification (Tomotada et al., 2003). Mori et al. (2004) designed an assay to monitor LAMP products in real-time by measurement of turbidity or fluorescence with a real-time thermal cycler or a real-time turbidimeter.

Given the great importance of the detection, identification and surveillance of *R. solanacearum* phylotype I mulberry strains in BWM integrated management, and that there have been no simple, rapid and sensitive LAMP-based methods available, we designed a subspecies-specific LAMP method targeting the MG67 DNA fragment which is generated from suppressive subtractive hybridization and proved to be well conserved in phylotype I mulberry strains (Pan et al., 2013; Cao et al., 2015). And we further evaluated the method on naturally infected mulberry samples in this study.

## Materials and Methods

### Strains Used in This Study

A total of 53 bacterial strains including 46 *R. solanacearum* strains and 7 other control bacteria strains were used in this study, and the characteristics of all strains are briefly presented in **Table 1**.

**Table 1:** *Ralstonia solanacearum* and the control strains used in this study.

No.	Strains	Host	Origin	Phylotype
***Ralstonia solanacearum***			
1	M2	Mulberry	Guangdong	I
2	M3	Mulberry	Shipan, Guangdong	I
3	M4	Mulberry	Shipan, Guangdong	I
4	M7	Mulberry	Shipan, Guangdong	I
5	M9	Mulberry	Shipan, Guangdong	I
6	M10	Mulberry	Zhejiang	I
7	M11	Mulberry	Zhejiang	I
8	M12	Mulberry	Tonglu, Zhejiang	I
9	M13	Mulberry	Tonglu, Zhejiang	I
10	M14	Mulberry	Tonglu, Zhejiang	I
11	M15	Mulberry	Tonglu, Zhejiang	I
12	M16	Mulberry	Tonglu, Zhejiang	I
13	M17	Mulberry	Tonglu, Zhejiang	I
14	M18	Mulberry	Tonglu, Zhejiang	I
15	M19	Mulberry	Tonglu, Zhejiang	I
16	M20	Mulberry	Linan, Zhejiang	I
17	M21	Mulberry	Linan, Zhejiang	I
18	M22	Mulberry	Linan, Zhejiang	I
19	M23	Mulberry	Linan, Zhejiang	I
20	M24	Mulberry	Linan, Zhejiang	I
21	M25	Mulberry	Linan, Zhejiang	I
22	M26	Mulberry	Linan, Zhejiang	I
23	M27	Mulberry	Linan, Zhejiang	I
24	M5	Mulberry	Shunde, Guangdong	I
25	M6	Mulberry	Shipai, Guangdong	I
26	Sn1	Night shade	Jinjiang, Fujian	I
27	Tb-Bs-1	Tobacco	Baise, Guangxi	I
28	GMI1000	Tomato	France	I
29	Bd1	Hibiscus	Putian, Fujian	I
30	Bp-Gk-1	Balsam pear	Guangxi	I
31	Ey-Aq-1	Eucalyptus	Guilin, Guangxi	I
32	Tm2	Tomato	Sanmenjiang, Guangxi	I
33	E1	Eggplant	Jinjiang, Fujian	I
34	Ssp1	Sesame	Liangfeng, Guangxi	I
35	R419	Banana	–	II
36	R264	Banana	–	II
37	R454	Banana	–	II
38	Po82	Potato	Mexico	II
39	Po35	Potato	–	III
40	Po40	Potato	Guangzhou, Guangdong	III
41	Po41	Potato	Peng county, Sichuan	II
42	PoYN	Potato	Yunnan	III
43	Z-Aq -1	Ginger	Anqiu, Shandong	I
44	Z-Rc-1	Ginger	Chongqing	I
45	Kp-1	Kaempferia panduratum	Guangxi	I
46	Z-Sch-1	Ginger	Sichuan	I
**Controls**			
47	*Enterobacter mori* Rs18-2	Mulberry	China	
48	*Erwinia carotovora* E12-1	Potato	China	
49	*Ralstonia mannitolilytica*	Soil	–	
50	*Ralstonia pickettii*	Soil	–	
51	*Enterobacter* sp	Soil	–	
52	*Acidovorax citrulli*	Watermelon	–	
53	*Burkholderia cepacia*	Onion	–	


*“–” means unknown.*

### DNA Preparation

The LAMP assays were evaluated on 53 bacterial strains. All strains were maintained in sterile distilled water at room temperature, streaked onto TZC agar (nutrient agar supplemented with 0.05% tetrazolium chloride) (Kelman, 1954), and incubated at 28°C for 48 h. Single slimy, milky colonies with a pink center were transferred to nutrient agar (0.3% beef extract, 1.0% glucose, 0.5% peptone, 0.05% yeast extract, and 1.8% agar) at 28°C for 48 h. Purified genomic DNAs were isolated with a commercial kit (Genomic DNA Purification kit; BioTeKe, Beijing, China) according to the manufacturer’s instructions. DNA concentrations were quantified photometrically (absorbance measurements at 260 and 280 nm, with a NanoVue Plus spectrophotometer; NanoDrop Technologies, USA).

### Primer Design

**FIGURE 1:**
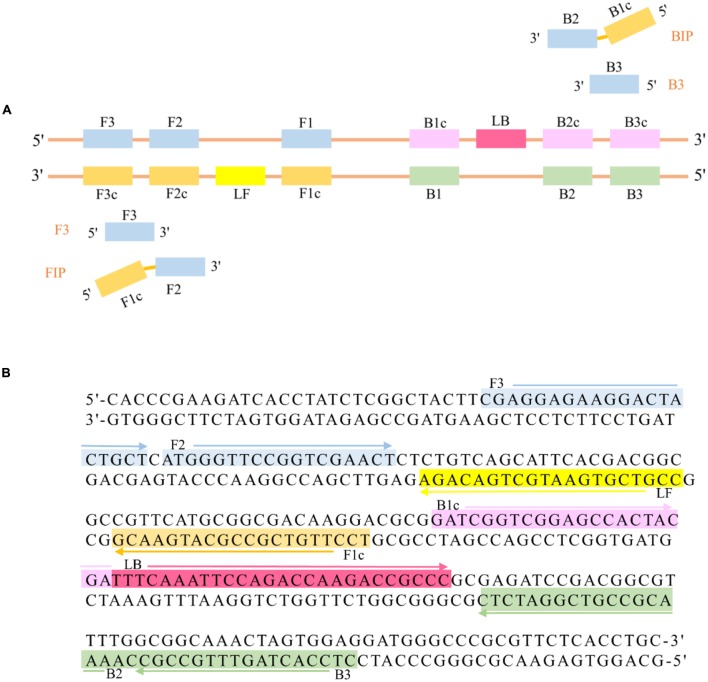
**(A)** Schematic diagram of two inner primers (FIP, BIP), two outer primers (F3, B3), and two loop primers (LF, LB) for LAMP. **(B)** *MG67* target DNA fragment. This sequence was used to design three pairs of primers, which are shown in different colors, with the arrows showing the orientation of the primers.

The LAMP primers (**Figure 1**) of the *R. solanacearum* phylotype I mulberry strains were designed targeting the gene *MG67* obtained from GenBank (accession number GS923075) and using the Primer Explorer V.4 software tool available on the Eiken Genome site^1^ provided by Eiken Chemical Co., Ltd. (Tokyo, Japan). The detailed sequences of the primers used for amplification of *R. solanacearum* phylotype I mulberry strains are shown in **Table 2**.

**Table 2:** Primer sequences for LAMP replication of the *MG67* gene.

Primer	Sequence
FIP	5′-TCCTTGTCGCCGCATGAACGATGGGTTCCGGTCGAACT-3′
BIP	5′-GATCGGTCGGAGCCACTACGAAAAACGCCGTCGGATCTC-3′
F3	5′-CGAGGAGAAGGACTACTGCT-3′
B3	5′-CTCCACTAGTTTGCCGCC-3′
LF	5′-CCGTCGTGAATGCTGACAGA-3′
LB	5′-TTTCAAATTCCAGACCAAGACCG-3′


### LAMP Reaction

Twenty five microliter of the LAMP reactions mixture contained 1.6 μM FIP and BIP, 0.2 μM F3 and B3, 0.4 μM LF and LB, 1.4 mM dNTPs, 1.0 M betaine (Sigma–Aldrich Corp, St. Louis, MO, USA), 20 mM Tris-HCl (pH 8.8), 50 mM KCl, 10 mM (NH_4_)_2_SO_4_, 8 mM MgSO_4_, 0.1% Tween-20, 8 U of the *Bst* DNA polymerase large fragment (New England Biolabs, USA), and 1 μl of the template DNA. Sterile deionized water was used as the template for the negative control.

These reactions were carried out in 0.2 ml microtubes, 1 μl of 1/10 diluted original SYBR Green I (Molecular Probes Inc.) was added in the inner lid of every microtube before incubating the reactions in a Veriti 96 well thermal cycler (Applied Biosystems, USA).

### Positivity Criteria and Running-Time Determination

Time of positivity (*T*_p_) and melting temperature (*T*_m_) are two basic parameters for positive signals. *T*_p_ is the time when the second derivative of fluorescent amplification reaches its peak above the baseline value, and this is expressed in minutes. The *T*_m_ is the temperature expressed in °C at which the amplification products melt into two single-stranded DNA molecules. In order to determine the optimal reaction temperature and time, we measured the turbidity of seven samples at different temperatures ranging from 60 to 65°C in real time.

ROX reference dye II (TaKaRa) (0.15 μl) and Eva Green (Biotium) (1.2 μl) were added to the LAMP reaction for the real-time LAMP. A real-time thermal cycler ABI 7500 (Applied Biosystems) with version 2.3 of the software was used to amplify and measure fluorescence at different reaction temperature and cycle. The program was: 1 s to react and 59 s to collect data for 50 cycles or more (1 cycle is 1 min) followed by the melting curve.

### Conventional Phylotype I Mulberry Strains-Specific Multiplex PCR

Phylotype I mulberry strains-specific primer set MG67-F/MG67-R, in combination with the species-specific primer pair AU759f/AU760r, was used to identify *R. solanacearum* phylotype I mulberry strains strains in a single PCR assay characterized by providing the expected amplicons of 156 and 282 bp (Pan et al., 2013). Each PCR mix had a total volume of 25 μl and contained 1× *Taq* MasterMix (PCR buffer, 1.5 mM MgCl_2_, 250 μM of each dNTP, 50 mM KCl, 10 mM Tris-HCl, and 1.25 U of *Taq* DNA polymerase; Tiangen Biotech), 6 pmoles of the primers MG67-F and MG67-R, and 4 pmoles of the primers AU759f and AU760r).

Initial denaturation was conducted at 94°C for 5 min, followed by 30 cycles of denaturation (30 s at 94°C), annealing (30 s at 60°C) and extension (30 s at 72°C), with a final extension step for 10 min. Subsequently, the PCR products were observed by being subjected to agarose gel electrophoresis.

### Verification of LAMP Detection Specificity

Fifty three representative strains that included 23 typical *R. solanacearum* phylotype I mulberry strains, 2 atypical *R. solanacearum* phylotype I mulberry strains, 21 *R. solanacearum* strains isolated from other plant species and 7 other soil-borne bacteria strains (*Enterobacter mori Rs18-2*, *Erwinia carotovora E12-1*, *R. mannitolilytica*, *R. pickettii*, *Enterobacter* sp., *Acidovorax citrulli*, and *Burkholderia cepacia*) were used to determine the specificity of the primers.

### Sensitivity of LAMP

In order to assess the sensitivity of the LAMP, serial 10-fold dilutions of the reference strain M7 pure culture and genomic DNA (at an initial concentration of 160 ng/μl) were prepared in sterile deionized water. A pure culture of M7 was plated on nutrient agar at 28°C for 48 h. The cell suspension from 2-day-old culture of M7 strain were adjusted to 10^8^ CFU/ml, and a series of decimal dilutions ranging from 10^8^ to 10^2^ CFU/ml were prepared. Genomic DNA was extracted as mentioned above and followed by 10-times gradient dilution.

Bacterial suspension or genomic DNA (1 μl) from each dilution series was added to the LAMP and conventional PCR reaction mixture, respectively, as the amplification template.

In addition, healthy mulberry leaves collected from Beijing were crushed in 10 ml of sterile deionized water and then mixed with newly prepared bacterial suspension of strain M7 to result in the following concentrations: 10^7^, 10^6^, 10^5^, 10^4^, 10^3^ CFU/ml, 10^2^, 10^1^ CFU/ml. A 1 μl sample of the resulting supernatant was then assayed using the LAMP method.

### Application of LAMP for Plant Samples

To demonstrate the field application of LAMP as a diagnostic tool for *R. solanacearum* phylotype I mulberry strains, 20 samples were collected from Tonglu district, Hangzhou city, in east China’s Zhejiang province, where is the epidemic areas. And 2 mulberry samples were collected from Beijing, where is the BWM-free areas.

The surfaces of stems or roots were flushed with clean water and disinfected with 70% ethyl alcohol. 1-cm-long plant tissues taken from these samples were cut into pieces with disinfected garden shears in sterile Petri dishes containing 10 ml of sterile deionized water, then suspended for 30 min. A total of 1 μl of each sample suspension was used as the amplification template for both the LAMP method and the conventional PCR.

## Results

### Visual Detection of the LAMP Reaction

**FIGURE 2:**
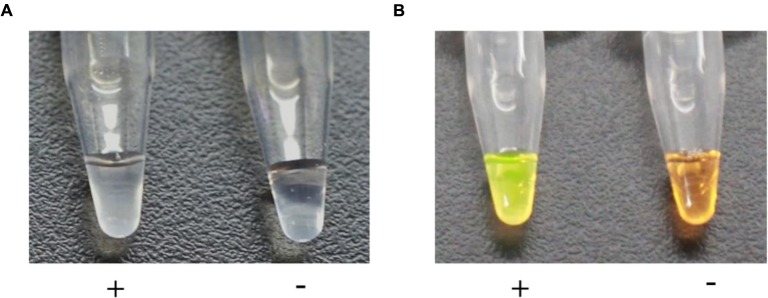
**Visual assessment of DNA amplification by LAMP.** **(A)** The turbidity indicating a positive reaction (+) was caused by the magnesium pyrophosphate precipitate produced during DNA synthesis, whereas the negative control (-) remained clear. **(B)** The solution turned green (+) in the presence of a LAMP amplification, while it remained orange (-) with no amplification.

One of the characteristics of LAMP is its ability to give results that can be identified with the naked eye due to the magnesium pyrophosphate precipitate produced during the reaction. We used 1 μl of genomic DNA of strain M7 as a template for the positive control and 1 μl of water as a template for the negtive control tn the LAMP assay. White turbidity could be seen in the positive reaction (+), whereas the negative control (-) remained clear (**Figure 2A**). A 1 μl aliquot of the 1/10 diluted original SYBR Green I was added to the solution of the positive and negative controls. After brief centrifugation, the positive control (+) turned green, while the negative control (-) remained orange (**Figure 2B**).

### Melting Curve Analysis

**FIGURE 3:**
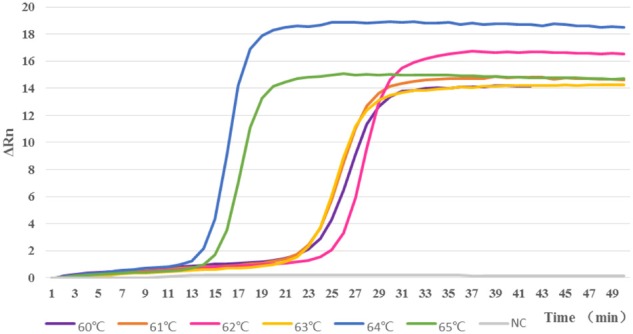
**Reactions were carried out at different temperatures from 60 to 65°C.** When tested at 64°C, the LAMP assay had the highest amplification efficiency, compared with the reactions at other temperatures.

In order to analyze the LAMP reactions in real-time, we used the genomic DNA of strain M7 as a template to perform real-time LAMP at given temperatures with a real-time thermal cycler ABI 7500. The real-time kinetics of the LAMP reaction were studied by monitoring fluorescence as described in Materials and Methods. The LAMP reactions were performed at different temperatures from 60 to 65°C; as shown in **Figure 3**, reaction at 64°C was the most productive as well as the most time-saving, and this amplification reached its peak in the 17th minute. Therefore, the LAMP reaction was finally determined to be best carried out at 64°C for 20 min.

### Specificity of LAMP

The specificity of the LAMP for detecting *R. solanacearum* phylotype I mulberry strains was analyzed using genomic DNA isolated from 46 representative *R. solanacearum* strains that belonged to Phylotypes I (sample 1–34; 43–46), II (sample 35–38; 41), and III (sample 39–40; 42), and 7 other pathogens, of which *Enterobacter mori* Rs18-2 (sample 47) and *Erwinia carotovora* E12-1 (sample 48) cause bacterial diseases in mulberry in China, *R. mannitolilytica* (sample 49) and *R. pickettii* (sample 50) are closely related to *R. solanacearum*, and *B. cepacia* (sample 51), *Enterobacter* sp. (sample 52) and *A. citrulli* (sample 53) are common soil-borne bacteria in nature.

**FIGURE 4:**
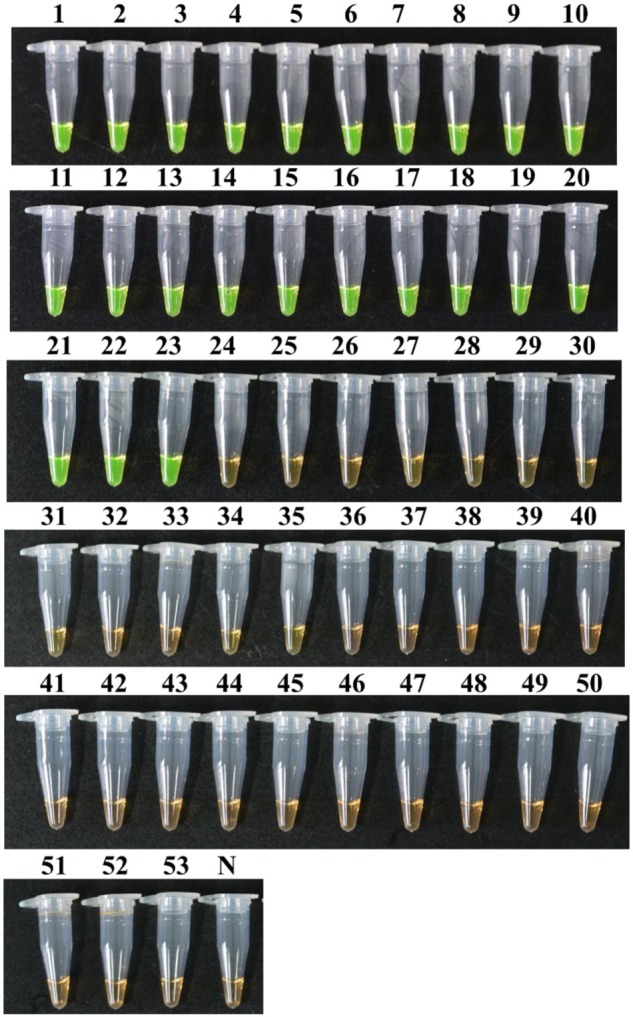
**Specificity of LAMP detection.** Tubes 1–53 correspond to strains 1–53 listed in **Table 1**. Tube N, negative control.

Positive reactions showed bright green fluorescence, while negative ones remained light orange after brief centrifugation. Amplifications were detected in all *R. solanacearum* phylotype I mulberry populations; in contrast, no amplifications were observed from other strains (**Figure 4**). This result is indicative that the primer set could be used to specifically detect *R. solanacearum* phylotype I mulberry strains. The conventional PCR with multiplex primers (MG67-F/MG67-R and AU759f/AU760r) also showed good specificity.

### Sensitivity of LAMP

**FIGURE 5:**
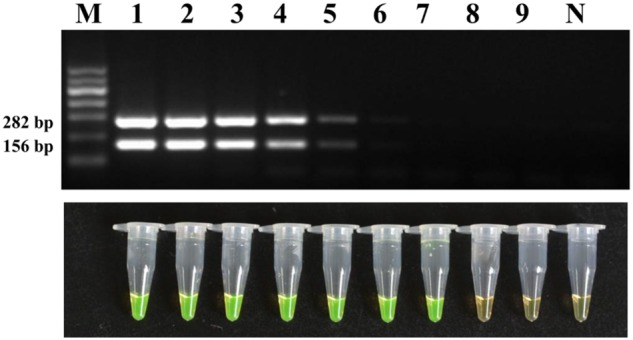
** Detection limit of the conventional PCR and LAMP method for DNA.** Tubes/lanes 1–9, nine different DNA concentrations made from a series of 10-fold dilutions: 160, 160 × 10^-1^, 160 × 10^-2^, 160 × 10^-3^, 160 × 10^-4^, 160 × 10^-5^, 160 × 10^-6^, 160 × 10^-7^, 160 × 10^-8^ ng/μl, respectively. Tube/lane N, negative control. Lane M, Marker I (700, 600, 500, 400, 300, 200, 100 kb) (BingDa Biotech).

The sensitivity was first evaluated with a 10-fold dilution series of genomic DNA of strain M7. The results (**Figure 5**) showed that LAMP could detect original, 10^-1^, 10^-2^, 10^-3^, 10^-4^, 10^-5^, and 10^-6^ diluent genomic DNA of strain M7, and the detection limit was 160 fg; while the conventional PCR could only detect original, 10^-1^, 10^-2^, 10^-3^, 10^-4^, and 10^-5^, diluent genome DNA of strain M7, which showed the sensitivity of the former was 10 times higher than that of the latter.

**FIGURE 6:**
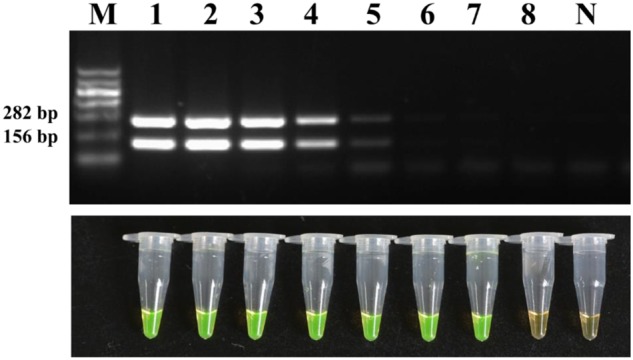
**Detection limit of the conventional PCR and LAMP method for the bacterial suspensions.** Tubes/lanes 1–8, eight different cell concentrations made from a series of 10-fold dilutions: 2.2 × 10^8^, 2.2 × 10^7^, 2.2 × 10^6^, 2.2 × 10^5^, 2.2 × 10^4^, 2.2 × 10^3^, 2.2 × 10^2^, 2.2 × 10^1^ CFU/ml, respectively. Tube/lane N, negative control. Lane M, Marker I (700, 600, 500, 400, 300, 200, 100 kb) (BingDa Biotech).

The sensitivity was then evaluated with a 10-fold dilution series of pure cultures of strain M7. The detection limit reached as low as 2.2 × 10^2^ CFU/ml of bacterial cells which is 100 times higher than the conventional PCR (**Figure 6**).

**FIGURE 7:**
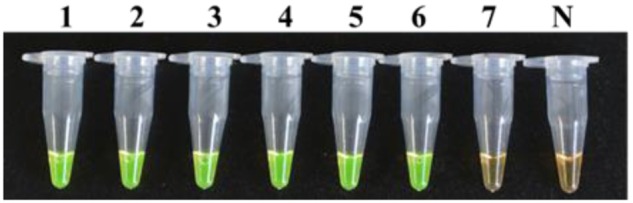
**Detection limit of the LAMP method for a dilution series of strain M7 with mulberry extracts.** Tubes 1–7, seven different dilutions made from a series of 10-fold bacterial suspensions of strain M7 with mulberry extracts: 2.2 × 10^7^, 2.2 × 10^6^, 2.2 × 10^5^, 2.2 × 10^4^, 2.2 × 10^3^, 2.2 × 10^2^, 2.2 × 10^1^ CFU/ml. Tube N, negative control.

The LAMP method could also detect 2.2 × 10^2^ CFU/ml of bacterial suspension of strain M7 mixed with the mulberry extract (**Figure 7**), which was indicative that the LAMP reaction was not affected by the presence of plant tissue fluid.

### Application of LAMP for Plant Samples

**FIGURE 8:**
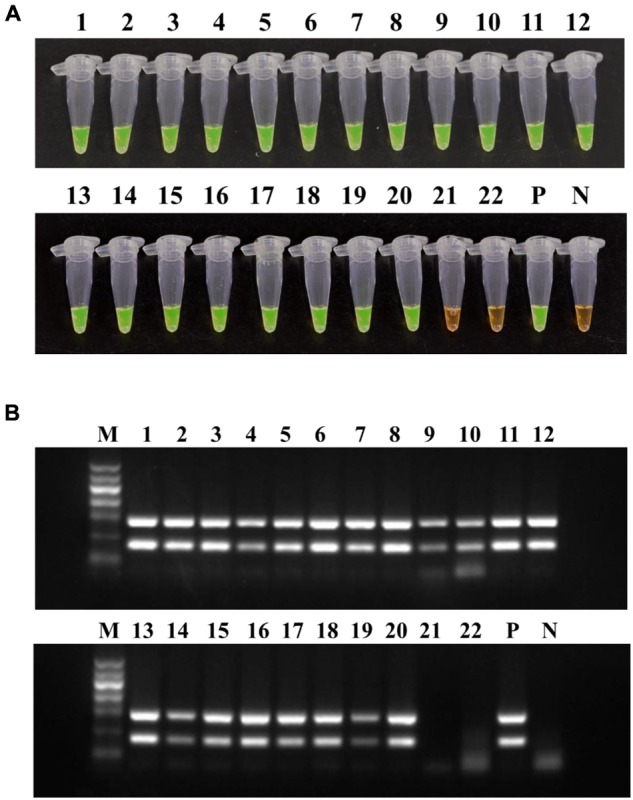
**Application of LAMP** **(A)** and conventional PCR **(B)** on mulberry samples. Tubes/lanes 1–20, mulberry samples collected from Zhejiang province. Tubes/lanes 21–22, mulberry samples collected from Beijing. Tube/lane P, positive control. Tube/lane N, negative control. Lane M, Marker I (700, 600, 500, 400, 300, 200, 100 kb) (BingDa Biotech).

The LAMP method and conventional PCR were performed on 22 mulberry samples collected from Zhejiang province and Beijing in China. LAMP and PCR were both positive for samples 1–20, for some of which obvious symptoms were observed. The non-infected samples (21–22) showed negative results (**Figure 8**). These results are indicative of the high detection capability of *R. solanacearum* phylotype I mulberry strains-specific LAMP.

‘

## Discussion

A plant disease early warning system based on rapid, sensitive and reliable detection of pathogens is the cornerstone of disease-management decision making. Up to now, numerous valuable cultures, serology, PCR and especially LAMP-based methods have been available for the detection of *R. solanacearum* at both the species and subspecies level (Kubota et al., 2008, 2011a,b; Lenarcic et al., 2014; Kubota and Jenkins, 2015). In contrast to the conventional PCR method, LAMP was suitable for on-site detection in the field because of its rapidity and robustness, and it could be applied even by laymen without the need of elaborate laboratory equipment (Kong et al., 2016).

*Ralstonia solanacearum* phylotype I mulberry strains, indigenous to China and undergoing lengthy coevolution with mulberry, constitutes a persistent menace to China’s sericulture industry. So far, however, no LAMP-based method for the detection of *R. solanacearum* phylotype I mulberry strains has been developed. Therefore, accurate identification of *R. solanacearum* at the infra-species level may be helpful to establish the most economically feasible crop rotation strategy.

In this study, to develop a highly practical and valid LAMP-based method for detecting *R. solanacearum* phylotype I mulberry strains, we designed a primer set consisting of six primers, in which F3, B3, FIP and BIP recognized six distinct sequences on the target DNA, *MG67*. LB and LF, the other two loop primers, which were designed for improving the detection efficiency, significantly shortened the reaction time from 70 to 18 min.

To evaluate the specificity of the LAMP-based method, 53 strains were used in this study, including 46 *R. solanacearum* strains covering Phylotypes I, II, III and seven other soil-borne bacteria strains. As expected, *R. solanacearum* phylotype I mulberry strains could be easily distinguished from the other strains, particularly the M5 and M6 that are atypical mulberry strains. The same results were observed with a multiplex PCR developed by Pan et al. (2013).

The LAMP method also showed high sensitivity over its PCR counterpart with the detection limit of 160 fg genomic DNA, which is 10 times higher than conventional PCR, and 2.2 × 10^2^ CFU/ml of bacterial cells, which is 100 times higher than conventional PCR. In addition, the sensitivity of the phylotype I mulberry strains-specific LAMP was higher than those of the LAMP assays of Kubota et al. (2008) and Lenarcic et al. (2014).

Since the LAMP reaction could produce many bands of different sizes, forming a ladder of DNA fragments of 100 bp and larger (Kubota et al., 2008), it is common to verify the amplification results with gel electrophoresis. However, the reaction products would easily cause aerosol pollution under high temperature conditions, which results in higher false positive rates. In addition, taking more time and energy for the gel electrophoresis makes the LAMP assay not suitable for application in the field. Lenarcic et al. (2014) used Genie II equipment to monitor the process of amplification. Although amplification processes were visible by this, the detection costs were greatly increased. A relatively simple approach to detect LAMP amplicons is observing the turbidity-change due to the formation of white precipitate, magnesium pyrophosphate (Mori et al., 2001; Tomotada et al., 2003). For better visibility of the reaction result, DNA intercalating dyes such as propidium iodide, ethidium bromide, methylene blue, acridine orange, and SYBR Green I have been added to reaction tubes (Hill, 2008); among them, SYBR Green I produced the best visual discrimination. Kubota et al. (2011b) developed a non-instrumented nucleic acid amplification method which can be potentially used in field for detection of *R. solanacearum* race 3-biovar 2 strains.

In this study, we added 1 μl of 1/10 diluted original SYBR Green I onto the inner lid of every centrifuge tube before the reaction. Since the color change depended on the amount of DNA, the solutions would turn green with the presence of the LAMP amplicons after brief centrifugation. In this way, not only aerosol pollution caused by opening the lids could be avoided but visual effect of LAMP detection could be improved as well. In addition, we could save more time and money. Compared to conventional phylotype I mulberry strains-specific PCR, the established LAMP-based method can be performed well in a Veriti 96 well thermal cyclera water bath, a heating block and even an insulated mug (AIJIABAO^®^, China), thus makeing it possible to be applied in on-site detection.

The applicability of the described LAMP-based assay was verified with a total of 22 field samples, collected from both BWM-free and epidemic areas, by a parallel test using multiplex PCR, plating isolation, and the LAMP method. The LAMP-based method showed significant advantages over its counterparts. Firstly, it was more efficient, taking less than an hour as compared with 3 h for PCR and several days for isolation. Secondly, it was more user-friendly, convenient and inexpensive, since either planting isolation or PCR can only be conducted on dedicated equipment in laboratories, whereas the phylotype I mulberry strains-specific LAMP assay can be run in any simple constant-temperature device, water or metal bath, or even a vacuum insulation cup; thereby the LAMP method has great potential to be used in the field. Lastly, due to the high population of *R. solanacearum* present in diseased mulberry samples, no difference in detection results were observed by using phylotype I mulberry strains-specific LAMP and conventional PCR. However, in the case of latently infected mulberry propagation material, if occur, in which phylotype I mulberry strains occurred in low titer, the more sensitive LAMP method could substantially reduce the risk of false negative results. The plating isolation only gave 16 positive results as a result of the overgrowth of saprophytic bacteria (data not shown). Nevertheless, conventional culture-based methods cannot be completely replaced by DNA-based methods in the plant- and food-related fields.

This is the first report on the application of the LAMP assay, as an alternative method, for the specific detection of *R. solanacearum* phylotype I mulberry strains.

## Conclusion

The LAMP-based method developed in this study allows for rapid and accurate identifications of *R. solanacearum* phylotype I mulberry strains. Due to its easy operation without sophisticated equipment, it could play an important role in the early diagnosis of BWM and provide technical support for disease-management decision making.

## Author Contributions

JX and JF designed this experiment and helped to collecting the plant samples. WH performed this experiment and wrote this manuscript. HZ and JX provided assistance during the experiment. JF, JX, SW, XK, and WD revised this manuscript.

## Conflict of Interest Statement

The authors declare that the research was conducted in the absence of any commercial or financial relationships that could be construed as a potential conflict of interest.

## Footnotes

1 http://primerexplorer.jp/e/

## Abbreviations

BIP: backward inner primer
BWM: bacterial wilt of mulberry
FIP: forward inner primer
LAMP: loop-mediated isothermal amplification
