# Corrigendum: Loop-Mediated Isothermal Amplification Method for the Rapid Detection of *Ralstonia solanacearum* Phylotype I Mulberry Strains in China

**DOI:** 10.3389/fpls.2017.00325

**Published:** 2017-03-09

**Authors:** Wen Huang, Hao Zhang, Jingsheng Xu, Shuai Wang, Xiangjiu Kong, Wei Ding, Jin Xu, Jie Feng

**Affiliations:** ^1^Institute of Plant Protection, Chinese Academy of Agricultural SciencesBeijing, China; ^2^College of Plant Protection, Southwest UniversityChongqing, China

**Keywords:** *Ralstonia solanacearum*, phylotype I mulberry strains, loop-mediated isothermal amplification, detection, bacterial wilt

There were mistakes regarding the phylotype of strain Po35, Po40, and PoYN. Those strains belong to Phylotype II, not Phylotype III.

In the Results section, sub-section Specificity, paragraph one, samples 35–38 and 41 were allocated to Phylotype II and samples 39–40 and 42 to Phylotype III. The correct paragraph should be:

The specificity of the LAMP for detecting *R. solanacearum* phylotype I mulberry strains was analyzed using genomic DNA isolated from 46 representative *R. solanacearum* strains that belonged to Phylotypes I (sample 1–34; 43–46), II (sample 35–42), and 7 other pathogens.

In addition, in the fourth paragraph of the Discussion, it was suggested that Phylotype III was also covered when it was not. The correct paragraph should be:

To evaluate the specificity of the LAMP-based method, 53 strains were used in this study, including 46 *R. solanacearum* strains covering Phylotypes I, II and seven other soil- borne bacteria strains.

Finally, in Table 1, the Phylotype of No. 39 (III), 40 (III), and 42 (III) are incorrect. The corrected table appears below.

**Table 1:** *****Ralstonia solanacearum*** and the control strains used in this study**.

**No**.	**Strains**	**Host**	**Origin**	**Phylotype**
***R. solanacearum***				
1	M2	Mulberry	Guangdong	I
2	M3	Mulberry	Shipan, Guangdong	I
3	M4	Mulberry	Shipan, Guangdong	I
4	M7	Mulberry	Shipan, Guangdong	I
5	M9	Mulberry	Shipan, Guangdong	I
6	M10	Mulberry	Zhejiang	I
7	M11	Mulberry	Zhejiang	I
8	M12	Mulberry	Tonglu, Zhejiang	I
9	M13	Mulberry	Tonglu, Zhejiang	I
10	M14	Mulberry	Tonglu, Zhejiang	I
11	M15	Mulberry	Tonglu, Zhejiang	I
12	M16	Mulberry	Tonglu, Zhejiang	I
13	M17	Mulberry	Tonglu, Zhejiang	I
14	M18	Mulberry	Tonglu, Zhejiang	I
15	M19	Mulberry	Tonglu, Zhejiang	I
16	M20	Mulberry	Linan, Zhejiang	I
17	M21	Mulberry	Linan, Zhejiang	I
18	M22	Mulberry	Linan, Zhejiang	I
19	M23	Mulberry	Linan, Zhejiang	I
20	M24	Mulberry	Linan, Zhejiang	I
21	M25	Mulberry	Linan, Zhejiang	I
22	M26	Mulberry	Linan, Zhejiang	I
23	M27	Mulberry	Linan, Zhejiang	I
24	M5	Mulberry	Shunde, Guangdong	I
25	M6	Mulberry	Shipai, Guangdong	I
26	Sn1	Night shade	Jinjiang, Fujian	I
27	Tb-Bs-1	Tobacco	Baise, Guangxi	I
28	GMI1000	Tomato	France	I
29	Bd1	Hibiscus	Putian, Fujian	I
30	Bp-Gk-1	Balsam pear	Guangxi	I
31	Ey-Aq-1	Eucalyptus	Guilin, Guangxi	I
32	Tm2	Tomato	Sanmenjiang, Guangxi	I
33	E1	Eggplant	Jinjiang, Fujian	I
34	Ssp1	Sesame	Liangfeng, Guangxi	I
35	R419	Banana	–	II
36	R264	Banana	–	II
37	R454	Banana	–	II
38	Po82	Potato	Mexico	II
39	Po35	Potato	–	II
40	Po40	Potato	Guangzhou, Guangdong	II
41	Po41	Potato	Peng county, Sichuan	II
42	PoYN	Potato	Yunnan	II
43	Z-Aq-1	Ginger	Anqiu, Shandong	I
44	Z-Rc-1	Ginger	Chongqing	I
45	Kp-1	Kaempferia panduratum	Guangxi	I
46	Z-Sch-1	Ginger	Sichuan	I
**Controls**				
47	*Enterobacter mori* Rs18-2	Mulberry	China	
48	*Erwiniacarotovora* E12-1	Potato	China	
49	*Ralstonia mannitolilytica*	Soil	–	
50	*Ralstonia pickettii*	Soil	–	
51	*Enterobacter* sp	Soil	–	
52	*Acidovorax citrulli*	Watermelon	–	
53	*Burkholderia cepacia*	Onion	–	

*“–” means unknown*.

The authors apologize for these errors and state that they do not affect the conclusion of the article in any way.

## Conflict of interest statement

The authors declare that the research was conducted in the absence of any commercial or financial relationships that could be construed as a potential conflict of interest.

