# Corrigendum: Loop-Mediated Isothermal Amplification Method for the Rapid Detection of *Ralstonia solanacearum* Phylotype I Mulberry Strains in China

**DOI:** 10.3389/fpls.2017.00560

**Published:** 2017-04-11

**Authors:** Wen Huang, Hao Zhang, Jingsheng Xu, Shuai Wang, Xiangjiu Kong, Wei Ding, Jin Xu, Jie Feng

**Affiliations:** ^1^State Key Laboratory for Biology of Plant Diseases and Insect Pests, Institute of Plant Protection, Chinese Academy of Agricultural SciencesBeijing, China; ^2^College of Plant Protection, Southwest UniversityChongqing, China

**Keywords:** *Ralstonia solanacearum*, phylotype I mulberry strains, loop-mediated isothermal amplification, detection, bacterial wilt

**Reason for Corrigendum:**

We forgot to add the State Key Laboratory for Biology of Plant Diseases and Insect Pests before the Institute of Plant Protection, Chinese Academy of Agricultural Sciences, Beijing, China.

**Correction:**

The affiliations of authors should be: ^1^State Key Laboratory for Biology of Plant Diseases and Insect Pests, Institute of Plant Protection, Chinese Academy of Agricultural Sciences, Beijing, China. ^2^College of Plant Protection, Southwest University, Chongqing, China.

The authors apologize for this error.

## Conflict of interest statement

The authors declare that the research was conducted in the absence of any commercial or financial relationships that could be construed as a potential conflict of interest.

